# CT and MR imaging features of mixed neuroendocrine–non-neuroendocrine neoplasm of the pancreas compared with pancreatic ductal adenocarcinoma and neuroendocrine tumor

**DOI:** 10.1186/s13244-023-01366-0

**Published:** 2023-01-24

**Authors:** Yanqi Zhong, Heng Zhang, Xian Wang, Zongqiong Sun, Yuxi Ge, Weiqiang Dou, Shudong Hu

**Affiliations:** 1 Department of Radiology, Affiliated Hospital, Jiangnan University, No. 1000, Hefeng Road, Wuxi, 214122 Jiangsu China; 2 Department of Radiology, Affiliated Renmin Hospital, Jiangsu University, No. 8, Dianli Road, Zhenjiang, 212002 Jiangsu China; 3 GE Healthcare, MR Research China, Beijing, 100176 China

**Keywords:** Pancreatic neoplasms, Mixed neuroendocrine–non-neuroendocrine neoplasm, MiNEN, Magnetic resonance imaging, Computed tomography

## Abstract

**Objective:**

This study aimed to assess the computed tomography (CT) and magnetic resonance imaging (MRI) features of pancreatic mixed neuroendocrine–non-neuroendocrine neoplasm (MiNEN) and compare them with those of pancreatic ductal adenocarcinoma (PDAC) and neuroendocrine tumor (NET).

**Methods:**

Twelve patients with pancreatic MiNEN, 24 patients with PDAC, and 24 patients with NET, who underwent both contrast-enhanced CT and MRI, were included. Clinical data and the key imaging features were retrospectively evaluated by two independent readers and compared between MiNEN and PDAC or NET. Univariate and multivariable logistic regression analyses were performed to obtain predictors for pancreatic MiNEN.

**Results:**

Patients with pancreatic MiNEN more frequently presented with large size and heterogeneous and cystic components compared with PDAC (*p* < 0.031) and ill-defined irregular margins, progressive enhancement, and adjacent organ involvement compared with NET (*p* < 0.036). However, vascular invasion was less commonly seen in MiNEN than PDAC (*p* = 0.010). Moderate enhancement was observed more frequently in MiNEN than in PDAC or NET (*p* < 0.001). Multivariate logistic analyses demonstrated that moderate enhancement and ill-defined irregular margin were the most valuable features for the prediction of pancreatic MiNEN (*p* ≤ 0.044). The combination of the two features resulted in a specificity of 93.8%, sensitivity of 83.3%, and accuracy of 91.7%.

**Conclusions:**

We have mainly described the radiological findings of pancreatic MiNEN with ill-defined irregular margin and moderate enhancement compared with PDAC and NET. The combination of imaging features could improve diagnostic efficiency and help in the selection of the correct treatment method.

## Key points

- Mixed neuroendocrine–non-neuroendocrine neoplasm is a rare pancreatic tumor (0.2%).
- Pancreatic MiNEN mainly presents as a relatively large heterogeneous mass invading adjacent organs in half of the cases.
- Ill-defined irregular margin and moderate enhancement are significant predictors.

## Introduction

Mixed neuroendocrine–non-neuroendocrine neoplasm (MiNEN) is commonly found in the stomach [1, 2], colon [3, 4], and nasopharynx [5]. According to the World Health Organization classification, MiNEN is defined as a heterogeneous tumor with two morphologically different neoplastic components, which include one neuroendocrine, and each component exceeds 30% of the tumor [6, 7]. The neuroendocrine part is always present [8]. As a very rare disease, it accounts for only 0.2% of all tumors in the pancreas [9].

Pancreatic MiNEN is a highly malignant tumor that exhibits variable clinical manifestations and poor prognosis with a five-year overall survival rate of 40% [10, 11]. The radiological and clinical features of MiNEN resemble those of neuroendocrine tumor (NET) or pancreatic ductal adenocarcinoma (PDAC), but the treatment for these three diseases differ, making differential diagnosis helpful to opt suitable treatment strategies for patients with pancreatic MiNEN [12].

Knowledge on MiNEN of the pancreas is limited because of the rarity of the disease and the lack of comprehensive studies. Previous studies have mainly focused on the clinicopathological features of pancreatic MiNEN and much less on radiological findings [11, 13, 14]. However, the preoperative diagnosis of mixed tumors remains challenging. The biopsy specimen might not be sufficient for the diagnosis of pancreatic MiNEN [15]. Computed tomography (CT) and magnetic resonance imaging (MRI) are routinely used to evaluate the characteristics of focal pancreatic lesions [16–18], but the imaging features of pancreatic MiNEN have not been studied in detail to differentiate it from PDAC or NET. Therefore, this study was performed to investigate the CT and MRI features of pancreatic MiNEN and compare them with those of PDAC and NET.

## Materials and methods

### Patient characteristics

This study was approved by our institutional review board, and patient informed consent was waived considering the retrospective nature of the study. From January 2015 to December 2020, 19 patients with pathologically confirmed pancreatic MiNEN were identified by searching medical database in our institution. Among these patients, 12 patients (male 8, female 4; mean age ± standard deviation [SD], 55.8 ± 11.1 years; range 37–73 years) were eventually enrolled in this study based on the following inclusion criteria: (1) CT and MR images included contrast enhancement (arterial phase and portal venous phase) before surgery, (2) no history of major abdominal surgery, and (3) primary pancreatic tumor. To form a 1:2 matching with the pancreatic MiNEN group, we selected 35 PDAC and 31 NET based on the above criteria. A total of 24 patients with PDAC (male 16, female 8; mean age ± SD, 58.9 ± 13.8 years; range 36–83 years) and 24 patients with pancreatic NET (male 16, female 8; mean age ± SD, 50.6 ± 14.5 years; range 31–72 years) were later selected based on gender, age, location, and treatment by using the Matchlt package of the R software (version 3.4.4, R Core Team 2017). All lesions were pathologically proven by pancreatectomy upon resection (*n* = 60).

### CT and MRI examination

#### CT examination

CT examinations were performed on 16 -or 64-multidetector CT scanners (Lightspeed 16 or Lightspeed 64; GE Medical Systems, Milwaukee, WI, USA) by using a dual-phase scan (arterial and portal phases). Arterial and portal phase scans were obtained with a delay of 15–20 s by using a bolus-tracking technique and a delay of 60–70 s after injection of 2.5–3 mL/kg of non-ionic iodinated contrast medium intravenously (Omnipaque 300 mg/mL, GE Healthcare) at a rate of 2.5–3.5 mL/s by using a power injector. The detector collimation values of GE 16- and 64-multidetector scanners were 0.75 and 0.6 mm, respectively, the pitch was 1.5, the rotation time was 0.6 s, the tube voltage was 120 kV, the tube current was 250 mA, the reconstructed slice thickness was 4.0 mm, and the reconstruction interval was 4.0 mm.

#### MRI examination

MRI examinations were performed using a 1.5-T MRI scanner (GE Signa HD, GE Healthcare Systems, Milwaukee, WI, USA) with 8- and 12-channel phased-array torso coil. The applied image sequences with scan parameters were shown as follows: repetition time (TR)/echo time (TE) of 520/14 ms, section thickness of 3 mm, field of view (FOV) of 400 × 280 mm^2^, and matrix of 256 × 256 for the fast-spin-echo-based pre-contrast T1-weighted images with fat suppression; (TR/TE) of 3,500/95 ms, section thickness of 3 mm, FOV of 400 × 280 mm^2^, and matrix of 320 × 256 for the respiratory-triggered fast-spin echo-based T2-weighted images with fat suppression; TR/TE of 4.9/1.0 ms, section thickness of 3 mm, FOV of 380 × 380 mm^2^, and matrix of 320 × 288 for the 2D-fast imaging employing steady-state acquisition (2D-FIESTA) in axial and coronal views; TR/TE of 3,600/70 ms, section thickness of 5 mm, FOV of 360 × 360 mm^2^, and matrix of 128 × 128 for the free-breathing single-shot echo-planar diffusion weighted image with *a* and *b* values of 0 and 800 s/mm^2^, respectively; and TR/TE of 4.1/1.5 ms, section thickness of 3 mm, FOV of 350 × 280 mm^2^, and matrix of 320 × 256 for the contrast-enhanced T1-weighted images with contrast-enhanced phases including arterial and portal phases. These parameters were applied at 15 s (arterial phase), 60 s (portal phase) after injection of Gadodiamide intravenously (Omniscan, GE; 0.1 mmol/kg body weight) at a rate of 1.5 mL/s by using an autoinjector.

### Imaging analysis

CT and MRI images were independently reviewed by two senior radiologists with 9 and 10 years of experience in abdominal imaging on the hospital image archiving and communication system. Interobserver agreement for imaging features was assessed after initial image analysis. Discrepancies between the two readers were resolved by a consensus after joint image re-evaluation. The paired CT and MRI data of 60 cases were randomly reviewed by two independent readers twice and the time interval between two assessments for each reader was least 1 month. Both readers were blinded to all clinical and pathological data.

The following lesion relevant characteristics were evaluated: largest transverse diameter of the tumor, tumor location, tumor contours and margins, heterogeneity, tumor calcification, presence of visible lymph nodes (short axis diameter > 10 mm), bile duct dilatation, presence of main pancreatic duct (MPD) enlargement (diameter > 3 mm), adjacent organ involvement, and vascular encasement by tumor. According to the internal components, tumors were classified as purely solid lesion, solid lesion with minor cystic components (cystic component < 10% of the tumor), mixed solid and cystic lesion (cystic component > 10% of the tumor) [19]. Areas with density and/or intensity similar to that of cerebrospinal fluid on both CT and MR images were considered as cystic components of tumor.

The enhancement characteristics on the arterial (pancreatic) phase include diffuse and rim type of increased enhancement. The enhancement characteristics on the portal venous phase include washout and progressive enhancement. The degree of CT enhancement was divided into no enhancement (+ 0–10 HU), mild (+ 10–20 HU), moderate (+ 20–50 HU), or marked (+ > 50 HU) enhancement compared with pre-contrast phase [20].

### Clinicopathological diagnoses

Available records of clinical and pathological data were retrieved for each patient. The following clinical data were extracted from medical records: sex, age, reasons for admission (abdominal pain or discomfort or jaundice), and surgical options. Tests for the serum tumor markers, including serum carcinoembryonic antigen (CEA), serum amylase, and serum carbohydrate antigen 19-9 (CA 19-9), were available in the clinical information system. These serological exams were mainly performed within 30 days before or after CT and MRI.

All sections were retrospectively reviewed by a pathologist with 16 years of experience, who was blinded to the imaging manifestations. The pathological diagnosis was based on hematoxylin–eosin and immunohistochemical staining results.

### Statistics analysis

Descriptive statistical values were calculated for all variables evaluated on CT and MRI. One-way ANOVA or Student’s t test was used for continuous variables, and chi-square test or Fisher’s exact test was used for categorical variables. These variables were then entered into univariate analysis with a conditional logistic regression model to determine the independent predictors of pancreatic MiNEN on CT and MRI. Multivariable logistic regression analyses were performed by adverse selection of significant variables in the univariable analyses combined with clinical significance, and intermediate factors were excluded. The sensitivity, specificity, accuracy, positive predictive value, negative predictive value (NPV), positive likelihood ratio (LR (+)), and negative likelihood ratio (LR (−)) of each significant imaging feature and combinations of these features were calculated.

Interobserver agreement was performed for each variable by using kappa (*k*) statistics with the following scale: poor, < 0.20; fair, 0.20–0.39; moderate, 0.40–0.59; substantial, 0.60–0.79; and almost perfect, 0.80–1.00 [21]. All statistical analyses were performed using SPSS Statistics 26.0 and R software (version 3.4.4, R Core Team 2017). Two-sided *p* values of < 0.05 were considered statistically significant.

## Results

### Clinical features

The clinical features of 60 patients are summarized in Table 1. Significant difference was observed in serum CA19-9 levels and surgical options among the three groups (*p* ≤ 0.015). No significant differences were observed in any other clinicopathological findings in Table 1. All cases were confirmed by postoperative pathological diagnosis.

**Table 1:** Clinicopathological characteristics of patients with pancreatic MiNEN, PDAC, and NET

Characteristic	MiNEN (*n* = 12)	PDAC (*n* = 24)	NET (*n* = 24)	*p* value^a^
Age (year)				
Mean ± SD	55.8 ± 11.1	58.9 ± 13.8	50.6 ± 14.5	0.331
Range	37–73	36–83	31–72	
Sex				> 0.999
Male	8	16	16	
Female	4	8	8	
Location				0.402
Body and tail	8	11	10	
Head and neck	4	13	14	
Clinical manifestation				–
Abdominal pain or discomfort	9	22	8	
Jaundice	2	4	0	
Serum CA19-9				0.015
Normal (< 35 U/mL)	8	13	22	
Elevated (35–994.2 U/mL)	4	11	2	
Serum amylase				> 0.999
Normal (40–110 U/L)	9	19	18	
Elevated (111–383 U/L)	3	5	6	
Surgery				< 0.001
Pancreaticoduodenectomy	3	12	6	
Distal pancreatectomy	7	6	8	
Local tumor resection	2	0	10	
Palliative surgery	0	6	0	

Continuous variables are expressed as the mean ± standard deviation, and categorical variables are described as the number of patients

*MiNEN*, mixed neuroendocrine–non-neuroendocrine neoplasm; *PDAC*, pancreatic ductal adenocarcinoma; *NET*, neuroendocrine tumor; *CA 19-9*, carbohydrate antigen 19-9

^a^Comparison among MiNEN, PDAC, and NET

### Unenhanced and contrast-enhanced CT and MRI imaging patterns

**Fig. 1:**
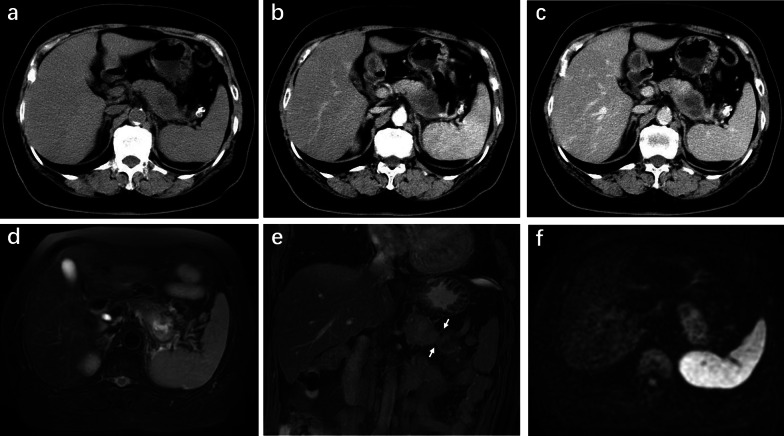
MiNEN of the pancreatic tail in a 63-year-old woman. **a** Unenhanced CT scan shows an ill-defined mass with a size of 8.0 cm. The CT value of the tumor is 39 HU. **b** Arterial phase CT scan shows peripheral moderate enhancement of the mixed solid and cystic lesion. The CT value of the tumor is 67 HU. **c** Portal phase CT scan shows heterogeneous enhancement with a central fill-in appearance. The CT value of the tumor is 75 HU. **d** Fat suppressed T2-weighted axial MR image indicates the hyperintense mass with significantly higher signal inside the lesion which was proven to be necrosis on histopathology. **e** Coronal fat suppressed 2D-FIESTA image shows a blurred interface between the tumor and the adjacent colon (arrows) which indicates that the fat filled gap disappears and the tumor infiltrates into surrounding intestinal wall. It is one typical sign of adjacent colon invasion which was confirmed by pathological results. **f** Diffusion-weighted MR image reveals relative hyperintense tumor indicating restricted diffusion

**Fig. 2:**
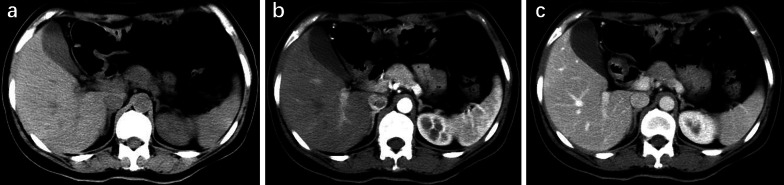
MiNEN of the pancreatic neck in a 48-year-old woman**. a** Unenhanced CT scan shows an ill-defined mass. The CT value of the tumor is 42 HU. **b** Arterial phase CT scan shows diffusely moderate homogeneous enhancement of the purely solid lesion with a size of 2.5 cm. The CT value of the tumor is 81 HU. **c** Portal phase CT scan shows washout of the oval tumor. The CT value of the tumor is 66 HU

In CT and MRI, pancreatic MiNEN lesions were located in the head and neck (*n* = 4) or body and tail (*n* = 8) of the pancreas (Table 1). The mean diameter of tumors was 4.7 ± 2.3 cm (range 2.5–9.0 cm), which was larger than that of PDAC (*p* = 0.031) and not statistically different from that of NET (*p* > 0.05) (Table 2). On unenhanced CT images, hypo-/iso-dense area was detected in all the 12 MiNEN lesions relative to the adjacent pancreatic parenchyma. Iso-/hyperintense signal was observed in all MiNEN lesions on T2-weighted MR images. Relative hyperintensity was observed in all 12 tumors on DWI-MRI. Representative cases of pancreatic MiNEN are shown in Figs. 1 and 2.

**Table 2:** Key imaging features observed on CT and MRI

	MiNEN, *n* (%) (*n* = 12)	PDAC, *n* (%) (*n* = 24)	NET, *n* (%) (*n* = 24)	*p* value^a^	*p* value^b^	*κ* value
Largest tumor diameter (cm)						
Mean ± SD	4.7 ± 2.3	3.0 ± 1.0	4.3 ± 2.3	0.031	0.589	0.98^c^
Range	2.5–9.0	1.4–7.0	1.0–12.0			
Tumor composition				0.003	0.722	0.74
Purely solid	4 (33.3)	20 (83.3)	9 (37.5)			
Minor cystic	3 (25)	3 (12.5)	3 (12.5)			
Mixed solid and cystic	5 (41.7)	1 (4.2)	12 (50)			
Heterogeneity	10 (83.3)	8 (33.3)	21 (87.5)	0.012	> 0.999	0.89
Tumor margin				0.588	< 0.001	0.9
Well-defined smooth	2 (16.7)	2 (8.3)	23 (95.8)			
Ill-defined irregular	10 (83.3)	22 (91.7)	1 (4.2)			
Enhancement degree				< 0.001	< 0.001	0.88
Mild	0 (0)	21 (87.5)	0 (0)			
Moderate	11 (91.7)	3 (12.5)	5 (20.8)			
Marked	1 (8.3)	0 (0)	19 (79.2)			
Arterial enhancement pattern				0.247	0.479	0.77
Rim type	5 (41.7)	5 (20.8)	7 (29.2)			
Diffuse type	7 (58.3)	19 (79.2)	17 (70.8)			
Portal enhancement pattern				> 0.999	0.031	0.91
Progressive enhancement	11 (91.7)	22 (91.7)	13 (54.2)			
Washout	1 (8.3)	2 (8.3)	11 (45.8)			
Tumor calcification	2 (16.7)	0 (0)	1 (4.2)	0.105	0.253	0.79
Visible lymph nodes	2 (16.7)	7 (29.2)	2 (8.3)	0.685	0.588	0.88
Bile duct dilatation	2 (16.7)	2 (8.3)	4 (16.7)	0.588	> 0.999	0.8
Marked upstream MPD dilatation	5 (41.7)	10 (41.7)	12 (50)	> 0.999	0.732	0.9
Adjacent organ involvement	6 (50)	13 (54.1)	3 (12.5)	> 0.999	0.036	0.82
Vascular involvement	3 (25)	18 (75)	3 (12.5)	0.01	0.378	0.76

Categorical variables are described as the number of patients with the percentage in parentheses

*CT*, computed tomography; *MRI*, magnetic resonance imaging; *MiNEN*, mixed neuroendocrine–non-neuroendocrine neoplasm; *PDAC*, pancreatic ductal adenocarcinoma; *NET*, neuroendocrine tumor; *MPD*, main pancreatic duct

^a^Comparison between MiNEN and PDAC; ^b^ comparison between MiNEN and NET; ^c^ Intraclass correlation coefficients with 95% CIs were calculated for largest tumor diameter

**Fig. 3:**
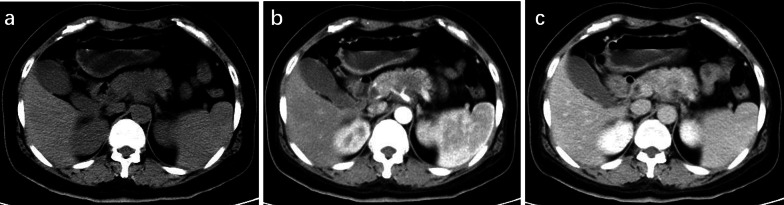
PDAC of the pancreatic body in a 51-year-old woman. **a** Unenhanced CT scan shows an ill-defined solid mass with a size of 4 cm. The CT value of the tumor is 36 HU. **b** Arterial phase CT scan shows diffusely mild homogeneous enhancement of the lesion and the celiac trunk is involved. The CT value of the tumor is 45 HU. **c** Portal phase CT scan show s progressively filling enhancement. The CT value of the tumor is 61 HU

**Fig. 4:**
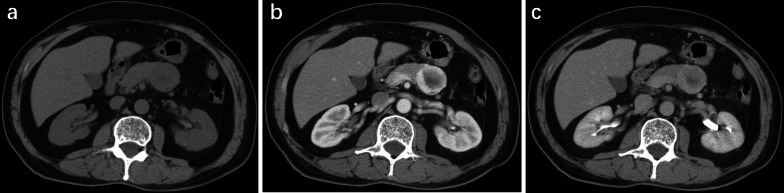
NET of the pancreatic body in a 72-year-old man. **a** Unenhanced CT scan shows a well-defined solid and cystic mass with a size of 3.5 cm. The CT value of the tumor is 41 HU. **b** Arterial phase CT scan shows marked heterogeneous enhancement of the lesion with a rim type. The CT value of the tumor is 133 HU. **c** Portal phase CT scan shows areas of washout. The CT value of the tumor is 98 HU

The comparison of the key imaging features of CT and MRI to differentiate pancreatic MiNEN from PDAC and NET is summarized in Table 2. In comparison with PDAC, pancreatic MiNEN more frequently presented with heterogeneous (83.3% vs. 33.3%, *p* = 0.012) and cystic components (66.7% vs. 16.7%, *p* = 0.003). In addition, ill-defined irregular margins (83.3% vs. 4.2%, *p* < 0.001), progressive enhancement (91.7% vs. 54.2%, *p* = 0.031), and adjacent organ involvement (50% vs 0.12.5%, *p* = 0.036) were more commonly observed in patients with pancreatic MiNEN compared with NET. Among them, the adjacent organ involvement of MiNEN included spleen in three cases, colon in two cases, and duodenum and bile duct in one case. However, vascular invasion was less commonly observed in MiNEN than PDAC (25% vs. 75%, *p* = 0.010). Moderate enhancement was observed more frequently in MiNEN than PDAC or NET (91.7% vs. 12.5% vs. 20.8%, *p* < 0.001) (Figs. 2, 3, and 4). The frequencies of the remaining features in Table 2 did not significantly differ among these groups (*p* ≥ 0.105). The interobserver agreement of imaging manifestation was substantial to almost perfect (*k* = 0.82–0.91 in terms of heterogeneity, tumor margin, enhancement degree, portal enhancement pattern, visible lymph nodes, marked upstream MPD dilatation, and adjacent organ involvement; *k* = 0.74–0.80 in terms of tumor composition, arterial enhancement pattern, tumor calcification, bile duct dilatation, and vascular involvement). The ICC for the largest tumor diameter between two observers was 0.98 (95% CI, 0.88–0.99, Table 2).

### Diagnostic efficacy of the key imaging features

Multivariable logistic regression analyses revealed that moderate enhancement relative to PDAC (*p* = 0.022) and ill-defined irregular margin relative to NET (*p* = 0.044) were independent predictors of pancreatic MiNEN (Table 3). The diagnostic performances on CT and MRI for these significant features and their combination are presented in Table 4. Among these parameters, moderate enhancement had the highest sensitivity (91.7%, 11/12), specificity (83.3%, 40/48), and accuracy (85%, 51/60) for the prediction of pancreatic MiNEN. In combination with tumor margin, the accuracy (91.7%, 55/60) and specificity (93.8%, 45/48) were generally higher, and the diagnosis is most likely MiNEN (LR (+) = 13.33). In addition, moderate enhancement resulted in the highest NPV (97.6%) and the lowest negative likelihood ratio (0.10).

**Table 3:** Results of univariate and multivariate conditional logistic regression analyses for differentiating pancreatic MiNEN from PDAC or NET

Variables	Univariable analysis			Multivariable analysis		
	OR	95% CI	*p* value	OR	95% CI	*p* value
*MiNEN versus PDAC*						
Tumor composition						
Mixed solid and cystic	0.1	0.01–0.86	0.036			
Heterogeneity	0.09	0.01–0.70	0.022	0.24	0.01–5.38	0.37
Moderate enhancement	0.05	0.01–0.41	0.005	0.08	0.01–0.69	0.022
Vascular involvement	4.65	1.22–17.65	0.024			
*MiNEN versus NET*						
Ill-defined irregular margin	0.05	0.01–0.39	0.004	0.1	0.01–0.94	0.044
Moderate enhancement	0.06	0.01–0.49	0.008	0.13	0.01–1.67	0.12
Portal progressive enhancement	0.12	0.01–1.02	0.052			
Adjacent organ involvement	0.19	0.04–0.95	0.043			

*MiNEN*, mixed neuroendocrine–non-neuroendocrine neoplasm; *PDAC*, pancreatic ductal adenocarcinoma; *NET*, neuroendocrine tumor; *OR*, odds ratio; *CI*, confidence interval

**Table 4:** Diagnostic performance of these significant features and combinations for predicting pancreatic MiNEN

	Sensitivity (%)	Specificity (%)	Accuracy (%)	PPV (%)	NPV (%)	LR (+)	LR (−)
*Imaging findings*							
Tumor composition							
Mixed solid and cystic	41.7 (5/12)	72.9 (35/48)	66.7 (40/60)	27.8 (5/18)	83.3 (35/42)	1.54 [0.68–3.47]	0.80 [0.49–1.31]
Heterogeneity	83.3 (10/12)	39.6 (19/48)	48.3 (29/60)	25.6 (10/39)	90.5 (19/21)	1.38 [0.98–1.94]	0.42 [0.11–1.59]
Portal enhancement pattern							
Progressive enhancement	91.7 (11/12)	27.1 (13/48)	40.0 (24/60)	23.9 (11/46)	92.9 (13/14)	1.26 [0.99–1.60]	0.31 [0.04–2.24]
Adjacent organ involvement	50.0 (6/12)	66.7 (32/48)	63.3 (38/60)	27.3 (6/22)	84.2 (32/38)	1.50 [0.75–3.00]	0.75 [0.42–1.35]
Vascular involvement	33.3 (4/12)	56.3 (27/48)	51.7 (31/60)	16.0 (4/25)	77.1 (27/35)	0.76 [0.32–1.80]	1.19 [0.77–1.83]
1. Tumor margin							
Ill-defined Irregular	83.3 (10/12)	52.1 (25/48)	58.3 (35/60)	30.3 (10/33)	92.5 (25/27)	1.74 [1.18–2.57]	0.32 [0.09–1.18]
2. Enhancement degree							
Moderate	91.7 (11/12)	83.3 (40/48)	85.0 (51/60)	57.9 (11/19)	97.6 (40/41)	5.50 [2.86–10.59]	0.10 [0.02–0.66]
1 + 2	83.3 (10/12)	93.8 (45/48)	91.7 (55/60)	76.9 (10/13)	95.7 (45/47)	13.33 [4.33–41.05]	0.18 [0.05–0.63]

Data are number of patients, unless otherwise indicated; data in parentheses are numerator/denominator of patients; data in square brackets are 95% CIs

*MiNEN*, mixed neuroendocrine–non-neuroendocrine neoplasm; *PPV*, positive predictive value; *NPV*, negative predictive value; *LR* (+), positive likelihood ratio; *LR* (−), negative likelihood ratio

## Discussion

Our study demonstrated that pancreatic MiNEN mainly showed as a large heterogeneous mass with ill-defined irregular margin, having moderate enhancement with progressive fill-in pattern. It mainly invaded adjacent organs, including spleen and colon rather than vessels. Among the parameters, ill-defined irregular margin compared with clear border of NET and moderate enhancement compared with mild enhancement of PDAC on CT and MRI were significant imaging predictors of pancreatic MiNEN. The combination of both features resulted in a high accuracy and specificity, thus allowing the identification of its classification and managing further treatment.

Among all the features, moderate enhancement had the highest specificity (83.3%, 40/48) for predicting pancreatic MiNEN. The degree of enhancement was measured by the dominant solid areas in the tumor due to the presence of cystic areas and heterogeneity [22]. Moderate enhancement could be clearly distinguished from PDAC (hypovascularity with progressive filling) or NET (hypervascularity with late and mild washout) [23–25]. Limited cases were available regarding the enhancement pattern of pancreatic MiNEN, but high-low mixed enhancement patterns have been reported, and similar results were obtained [11, 26]. Moreover, the enhanced imaging features of pancreatic MiNEN can be observed as a mixture of that of PDAC and NET.

Ill-defined irregular margin was a predictor of pancreatic MiNEN, which is similar to PDAC, but not same as clear border of NET [24, 27, 28]. Considering the high malignancy of MiNEN, the tumor may invade the boundary and extend further, which may cause ill-defined irregular margins. It is a clear implication that the tumor shows infiltrative desmoplastic features which implies surgical treatment when possible. As the tumor progresses, adjacent organs and tissue can be involved. Surprisingly, vascular involvement was significantly less frequent as that of PDAC, probably because it is not as erosive as PDAC. Nießen obtained the same findings [12].

MiNEN is manifested as mixed neoplasms with both endocrine and non-endocrine differentiation. Although both adenocarcinomas and pancreatic endocrine tumors are distinct entities, their components can be combined, resulting in heterogeneous pathological and morphological appearances. Previous case reports have described the imaging appearance of MiNEN as a heterogeneous cystic and solid mass, as observed in our study [26, 29].

A significant difference was observed in the largest tumor diameter between MiNEN and PDAC. Pancreatic MiNEN usually presents as a relatively large tumor [30, 31]. A possible explanation for this might be that pancreatic MiNEN rarely shows obvious symptoms resulting in late detection. By comparison, PDAC usually exhibits a mean diameter of 2–3 cm [32], as observed in our study.

Neither the role of surgery nor the effect of adjuvant chemotherapy and radiotherapy is clear in pancreatic MiNEN [33]. Once it is suspected, radical resection would be preferred [12]. However, in the present study, the surgery option of pancreatic MiNEN was not completely consistent with NET or PDAC. By contrast, PDAC resection is relatively extensive and NET conservative, while MiNEN is probably somewhere in between. Moreover, it is reported that MiNEN has a better prognosis than PDAC [12]. Therefore, CT and MR imaging features could be used as a pre-surgical clinical decision support tool to help in the differential diagnosis of pancreatic MiNEN, thus potentially influencing the surgical options and chemotherapy due to the different components. The results of the present study indicate the occurrence of pancreatic MiNEN when both two imaging features (ill-defined irregular margin and moderate enhancement) are present, whereas patients without either of the two features could be considered otherwise.

Our study has some limitations. First, the small sample size might limit identification of imaging features that may be suggestive of pancreatic MiNEN, although it reflects the rarity of this tumor type. Moreover, considering the retrospective, single-center nature of the study with an inherent selection bias as we only evaluated the resected tumors, our findings may not represent the full spectrum of pancreatic MiNEN.

In conclusion, we have mainly described the radiological findings of pancreatic MiNEN with ill-defined irregular margin and moderate enhancement compared with PDAC and NET. The combination of imaging features could improve the diagnostic efficiency and help in the selection of appropriate treatment.

## Data Availability

Please contact author for data requests.

## Abbreviations

CA19-9: Carbohydrate antigen 19-9
CEA: Carcinoembryonic antigen
CT: Computed tomography
DWI: Diffusion weighted imaging
LR: Likelihood ratio
MiNEN: Mixed neuroendocrine–non-neuroendocrine neoplasm
MPD: Main pancreatic duct
MRI: Magnetic resonance imaging
NET: Neuroendocrine tumor
NPV: Negative predictive value
PDAC: Pancreatic ductal adenocarcinoma
PPV: Positive predictive value
WHO: World Health Organization
